# Evaluation of the stray radiation distribution around a mobile cone beam computed tomography system in a simulated operating room environment

**DOI:** 10.1002/acm2.70434

**Published:** 2026-01-12

**Authors:** A. Kyle Jones

**Affiliations:** ^1^ Departments of Imaging Physics and Interventional Radiology Division of Diagnostic Imaging, MD Anderson Cancer Center Houston, TX USA

**Keywords:** image‐guided surgery, occupational dose, radiation protection, radiation scatter

## Abstract

**Background:**

Radiation protection in the operating room (OR) environment is a subject of much discussion in both the surgery and medical physics communities. Radiation exposure is often infrequent during image‐guided procedures, especially when only 3D imaging for navigation is used. This is accompanied by unique personnel considerations, including staff that rotate in and out of the OR and staff that are scrubbed in and do not have the opportunity to easily don and doff radioprotective garments. These communities seek clear guidance about the magnitude of stray radiation dose in the OR environment. However, prior studies have reported conflicting data on the topic and have used different methods and instruments.

**Purpose:**

To systematically measure the magnitude of stray radiation doses in a simulated OR environment in locations relevant to the placement of personnel during image‐guided spine surgery and to make recommendations for radiation protection based on these data when using a mobile cone beam computed tomography (CBCT) system and a realistic anthropomorphic phantom on an actual spine surgery table.

**Methods:**

Measurements of stray radiation dose were performed in a grid pattern in a simulated OR environment using pressurized ionization chamber survey meters and two configurations of a tissue‐mimicking anthropomorphic phantom, large (L) and extra‐large (XL). The phantom was imaged using “navigation” mode (i.e., CBCT) with standard and high definition (HD) protocols. Stray radiation dose was measured at heights corresponding to chest level (125 cm) and eye level (175 cm) of a typical operator and additional heights of 100 and 150 cm.

**Results:**

The absolute per scan whole body dose in the shadow of the gantry console 2 m from isocenter at a height of 125 cm (chest level) was 1.26 µSv for an equal mix of L and XL patients, and at a height of 175 cm (eye lens level) the air kerma was 9.22 µGy. The dose at 2 m from isocenter on the head side of the patient at a height of 125 cm was 7.93 µSv and air kerma at a height of 175 cm was 14.7 µGy. Doses at the same distance from isocenter and same heights on the side of the gantry opposite the console were 13.5 µSv and 15.0 µGy.

**Conclusions:**

Stray radiation doses were lowest in the shadow of the gantry console and were higher at a height of 175 cm compared to a height of 125 cm. Based on measured stray radiation doses at a distance of 2 m from isocenter, multiple radiation protection strategies can be employed to maintain occupational doses as low as reasonably achievable for operating room personnel.

## 1 INTRODUCTION

Radiation scatter is a frequently discussed topic related to intra‐operative volumetric imaging, especially as it relates to personnel placement during imaging. The stray radiation distribution in the operating room (OR) environment is affected by the imaging equipment, the imaged object, the imaging protocol, and the location of the equipment and personnel within the OR.

Although the stray radiation distribution is characteristic of the imaging equipment, it may change with beam energy and beam width or scan field of view. Therefore, the primary radiation protection considerations are the placement of personnel within the room and any radiation protection equipment used, including mobile shields and protective garments. Knowledge of the detailed scatter distribution aids in determining optimal placement of personnel within the room during volumetric imaging.

Manufacturers of medical imaging equipment provide plots of stray radiation distribution, typically for a single set of exposure conditions using a standard phantom. However, this does not match the clinical environment in which the equipment is used, with patients who are much longer than phantoms typically used to produce scatter isokerma plots and surgical tables that attenuate radiation scattered by the patient.

Occupational radiation exposure is one of many hazards in the OR environment. Most of these hazards are outside the scope of this work, but two are related – occupational radiation dose and orthopedic strain. Wearing personal radioprotective garments for long periods of time has been linked to an increased risk of orthopedic and neurological injury^1^, ^2^, ^3^. However, opportunities for personnel to don and doff radioprotective garments is limited when they are scrubbed in for surgical procedures.

The aim of this study was to perform a comprehensive assessment of the stray radiation distribution around a mobile cone beam computed tomography (CBCT) system in a simulated OR environment and make recommendations for radiation protection based on the acquired data. The assessment performed in this study used two sizes of a complete anthropomorphic phantom, imaging protocols with different baseline doses and fields of view, radiation detectors well‐suited to the data collected, a realistic simulated OR environment, and sampled the stray radiation distribution with up to 50 cm spatial resolution.

## 2 METHODS

**FIGURE 1:**
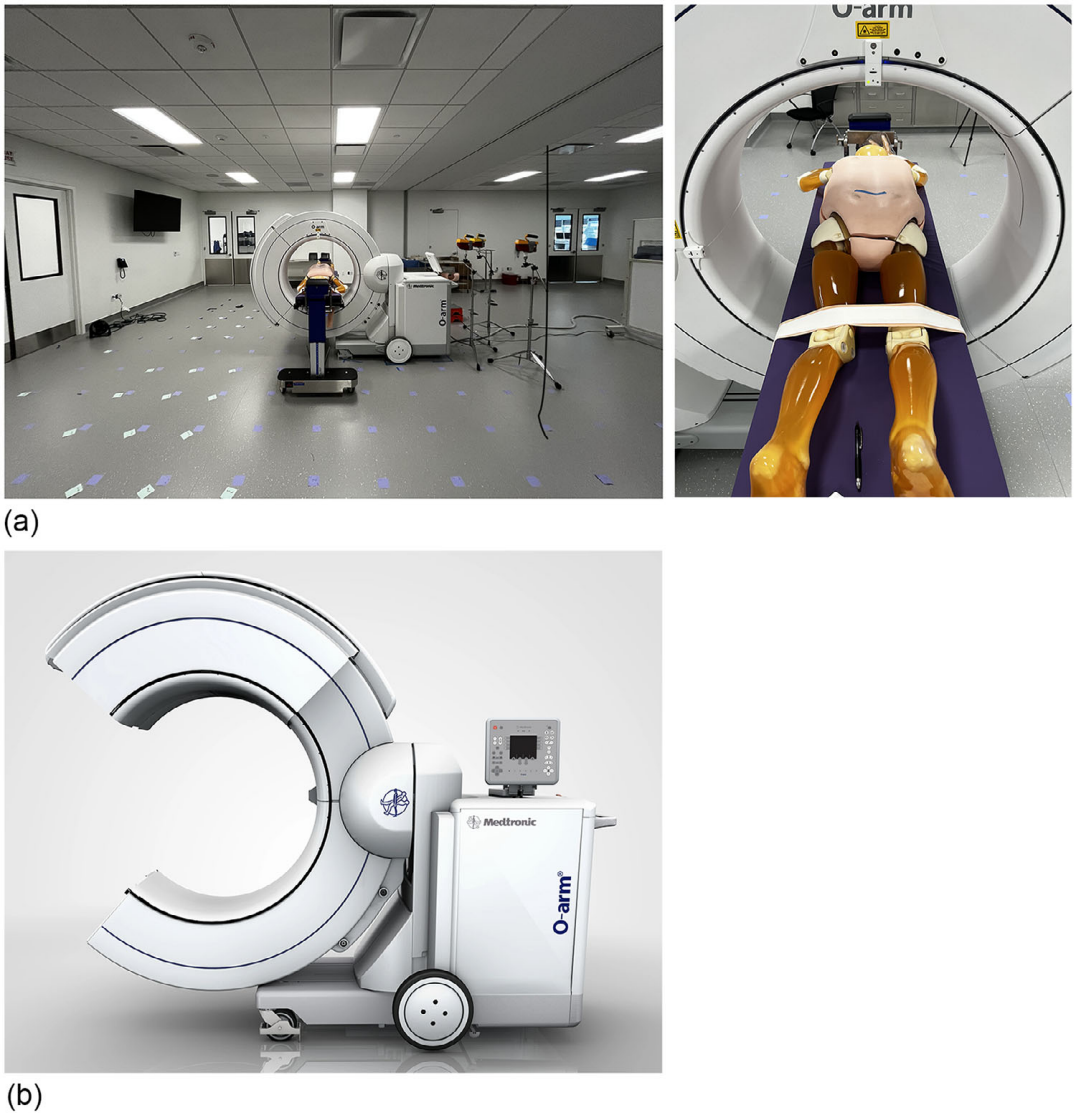
(a) Experimental setup. The phantom is in the L patient configuration with adipose tissues slabs to achieve a BMI of 32. (b) Illustration of gantry in open position (used by permission).

The mobile CBCT imaging system evaluated (O‐arm surgical imaging system, O‐arm O‐2 Image Acquisition System, Medtronic, Minneapolis, MN) was running software version 4.2.1. An anthropomorphic phantom (PBU‐50, Kyoto Kagaku, Kyoto, Japan) was imaged on an Advance Spine Table (Hillrom, Chicago, IL) in an OR simulation room with dimensions 10.4 m × 8.5 m. The phantom weighted 50 kg, was 165 cm long, and was constructed of resins and epoxies designed to mimic the radiation attenuation properties of human tissues. It was imaged in two configurations, both with “BMI plates” (adipose tissue slabs) produced by the manufacturer – a large (L) patient (body mass index [BMI] = 32) and an extra‐large (XL) patient (BMI = 40). The protocols used to image the two simulated patient sizes are described in Table 1 and the simulation setup is detailed in Figure 1. The surgical table was positioned at a working height of 89.5 cm from floor to tabletop, and the patient phantom was positioned at isocenter using anteroposterior and lateral fluoroscopy to image an area from L1 to L5.

**TABLE 1:** Protocols used to scan the simulated patients.

Body region	Patient size	Dose level	kV	mA	mAs	Axial FOV
Abdomen	L	Standard	120	80	320	20 cm
Abdomen	XL	HD	140	62	472	40 cm

**FIGURE 2:**
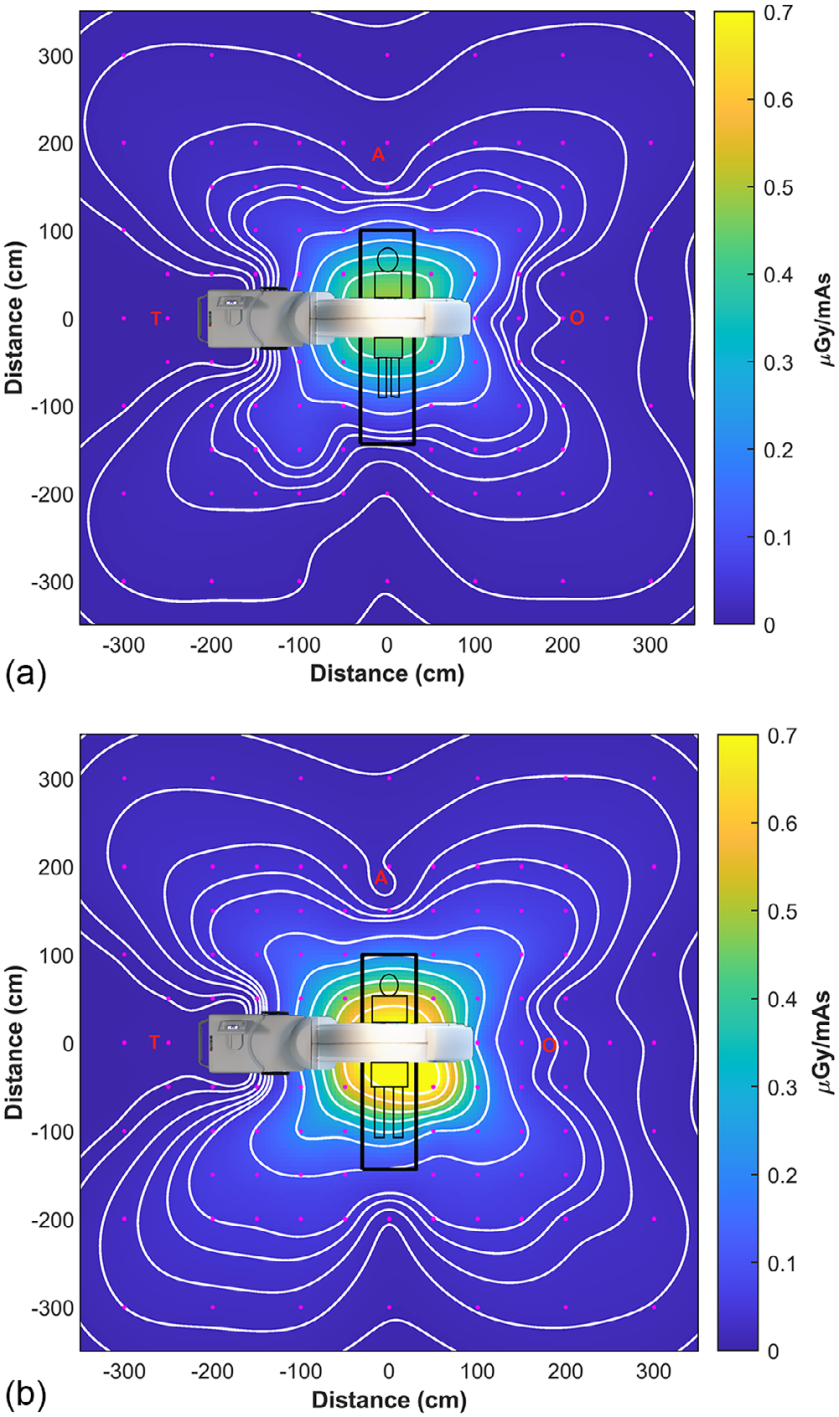
Measured stray radiation distribution for the (a) L phantom and (b) XL phantom. Measurement points for stray radiation dose at a height of 125 cm are represented by the magenta points in the figures. “T” represents the typical location of the radiologic technologist in the shadow of the gantry console, “A” represents the typical location of anesthesia, and “O” denotes the side of the gantry that opens, opposite the console. Contour lines from outside in represent doses of (a) 0.005, 0.01, 0.02, 0.03, 0.04, 0.05, 0.1, 0.2, 0.3, 0.4 µGy/mAs and (b) 0.01, 0.02, 0.03, 0.04, 0.05, 0.1, 0.2, 0.3, 0.4, 0.5, and 0.6 µGy/mAs.

Baseline calibration of the mobile CBCT system was verified by performing CTDI_vol_ measurements according to the manufacturer's instructions at 120 and 140 kV using a 32 cm CTDI phantom. Three survey meters (451P‐RYR, Fluke, Everett, WA, calibrated to Cs‐137 within the prior year) were used in integrate mode for stray radiation measurements, and readings in mR were multiplied by 8.76 to calculate air kerma (K_a_) in µGy. Reproducibility was assessed by repeating one measurement four times using the same survey meter and the same measurement with each of the three different survey meters. Measurements were made across a 3 m^2^ cartesian grid with a resolution of 50 cm out to 2 m (3 m in the shadow of the gantry on each side) and a resolution of 1 m from 2 m to 3 m (Figure 2). Measurement heights were referenced to an individual of height 178 cm, with chest level being 125 cm and eye level 175 cm. Additional measurements were acquired at heights of 100 and 150 cm to profile the stray radiation distribution along the table and gantry axes. No correction was applied for environmental background dose rates as each measurement was completed in under 10 s.

Contour plots of stray radiation were generated using Matlab R2023b (Natick, MA). Measured data were interpolated onto a grid of resolution 5 cm using a biharmonic spline (“griddata” function, method “v4”).

## 3 RESULTS

CTDI_vol_ measured according to manufacturer instructions was 12.9 % and 9.9% lower than the nominal values in the dosimetry report of the User's Manual^4^ at 120 and 140 kV, respectively. This is within the tolerance of ±20% recommended by the report of American Association of Physicists in Medicine (AAPM) Task Group 233^5^ and the ±40% tolerance specified by the manufacturer. The coefficient of variation of measurements in this study was 4.6%, the specified accuracy of the 451P‐RYR is ±10%,^6^ and the energy dependence of the 451P‐RYR is approximately −25% to +10% for the experimental setup used,^6^, ^7^ which yields an expanded uncertainty of approximately −27% to +15% for the stray radiation doses reported in this study.

**FIGURE 3:**
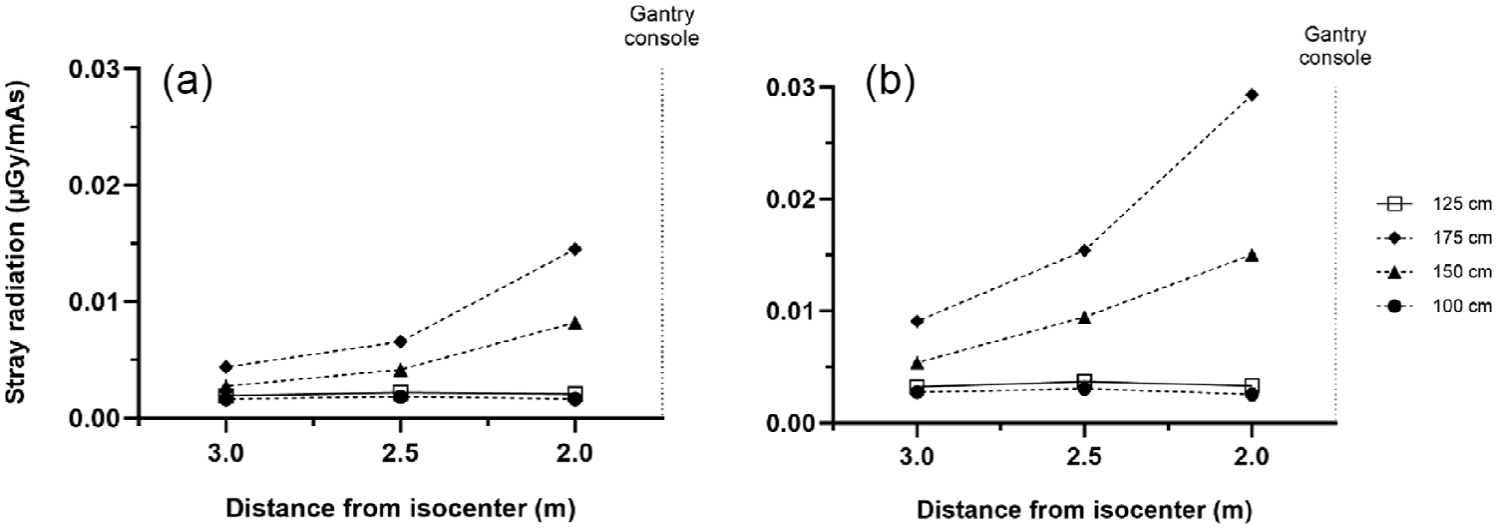
Measured stray radiation distribution at four heights at location T for the (a) L phantom and (b) XL phantom.

The measured stray radiation distribution for both the L and XL patients had a clover leaf shape (Figure 2), with higher intensities of stray radiation measured when imaging the XL patient. As expected, in most areas the intensity of stray radiation decreased with the square of the distance from isocenter. However, in the shadow of the gantry on the console side (location “T”) the intensity of stray radiation was very low and constant (Figures 2a and 3).

**FIGURE 4:**
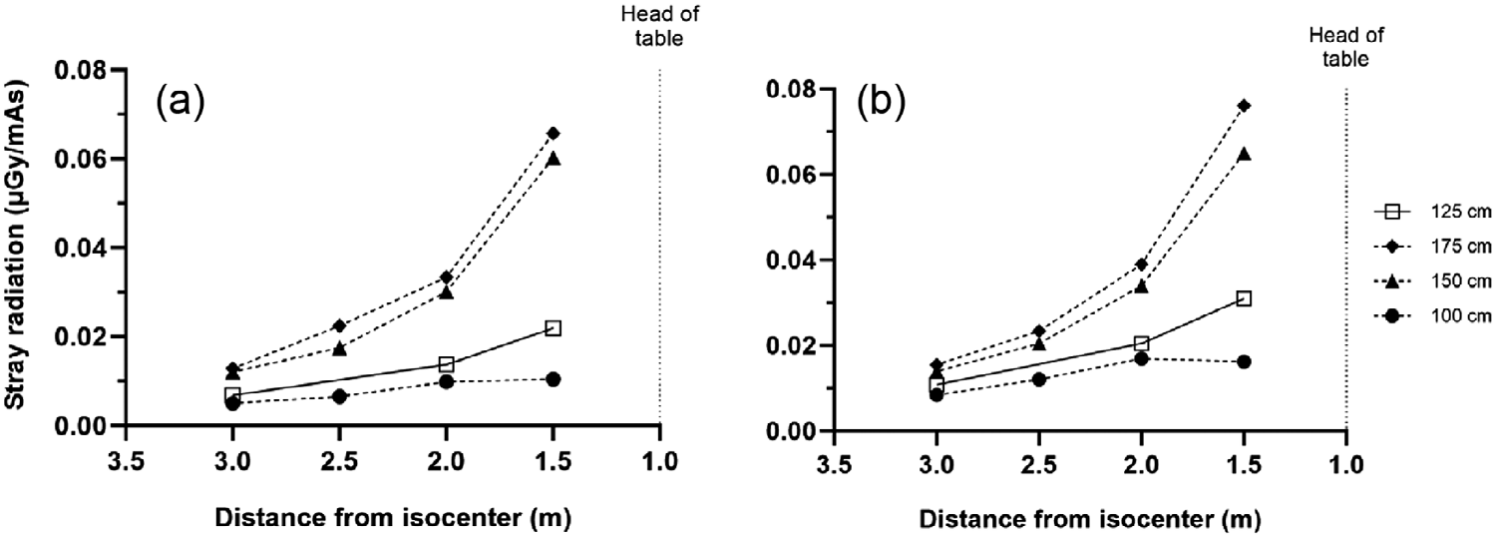
Measured stray radiation distribution at four heights at location A for the (a) L phantom and (b) XL phantom.

**FIGURE 5:**
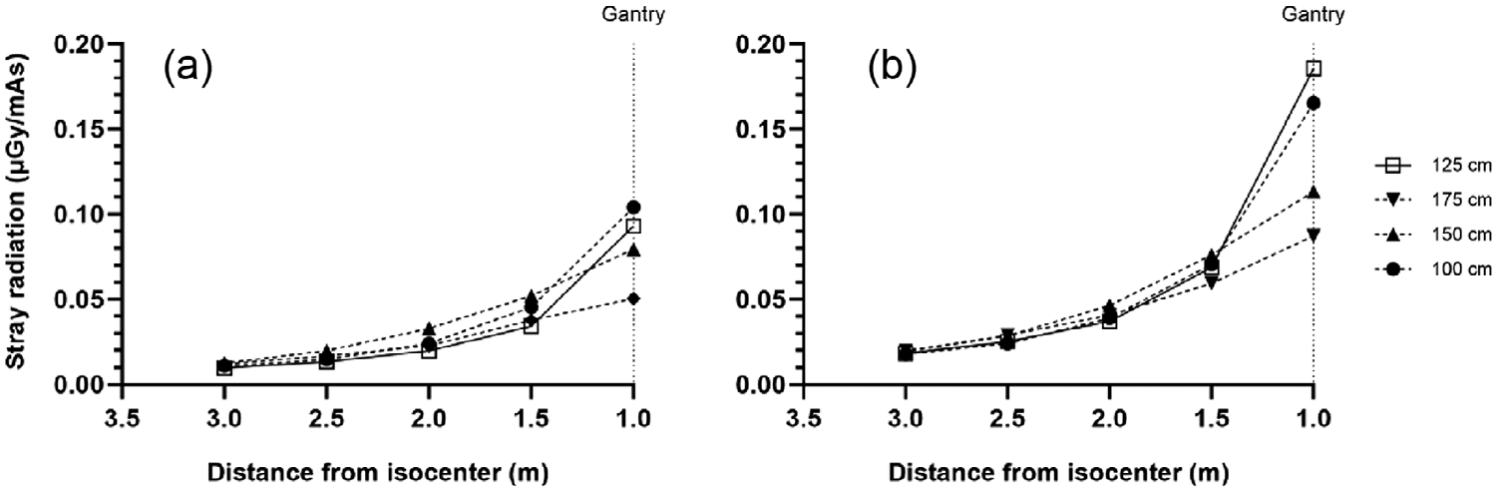
Measured stray radiation distribution at four heights at location O for the (a) L phantom and (b) XL phantom.

Figures 3, 4, 5 plot the intensity of stray radiation at different heights at location T (Figure 3), A (Figure 4), and O (Figure 5). At location T, stray radiation intensity was very low but increased with increasing measurement height (Figure 3). The falloff was less steep than expected based on the inverse square law and the intensity was constant at heights of 125 and 100 cm. Similar results were observed at location A (Figure 4), however, stray radiation intensity was more similar for the L and XL phantoms. The trend at heights of 175 and 150 cm was closer to pure inverse square law, and while stray radiation intensity decreased with distance from isocenter at heights of 125 and 100 cm, the decrease was closer to linear. Stray radiation intensity measured at location O was similar to that measured at the same distance from isocenter at A, however, there was little difference in stray radiation intensity at different heights in this direction (Figure 5).

Absolute stray radiation K_a_ rates at location T were very low and corresponded to per‐scan K_a_ at a height of 125 cm of 0.66 µGy for the L patient and 1.57 µGy for the XL patient (Table 1). The operational quantities ambient dose equivalent [H*(10)] and personal dose equivalent [H_p_(10)], which are equivalent for photons with energy ≤ 6 MeV,^8^ can be estimated by multiplying these K_a_ values by 1.13.^8^ This was determined based on previous work on scatter mimicking primary beam (SMPB) qualities for computed tomography (CT)^7^, ^9^ which demonstrated that an SMPB for scatter from 120 kVp primary radiation in CT can be produced using a primary beam energy of 80 kVp with 7.65 mm added aluminum (Al) and 8.82 mm added polymethylmethacrylate filtration. This SMPB has a half‐value layer of 5.75 mm Al. These characteristics are very similar to the N‐80 beam quality from International Standards Organization report ISO 4037‐1:2019^10^, which is included in the beam qualities for which Otto calculated H_p_(10) for different exposure geometries.^8^ The factor of 1.13 is derived by averaging H_p_(*S*, 0°), 1.37, and H_p_(*S*, ROT), 0.89, the spectrum‐averaged coefficients for anteroposterior irradiation and rotationally‐symmetric radiation, respectively, which is representative of the scenario simulated in this work. This yields per‐scan H_p_(10) of 0.75 and 1.77 µSv for the L and XL patient, respectively (Table 2).

**TABLE 2:** Per scan K_a_ and stray radiation doses at 2 m from isocenter. Stray radiation doses are in parentheses when reported.

	Location T		Location O		Location A	
	Chest (125 cm)	Eye (175 cm)	Chest (125 cm)	Eye (175 cm)	Chest (125 cm)	Eye (175 cm)
L phantom	0.66 µGy (0.75 µSv)	4.64 µGy	6.31 µGy (7.13 µSv)	10.7 µGy	4.38 µGy (4.95 µSv)	10.9 µGy
XL phantom	1.57 µGy (1.77 µSv)	13.8 µGy	17.5 µGy (19.8 µSv)	19.3 µGy	9.64 µGy (10.9 µSv)	18.4 µGy
Average for equal mix of L/XL patients	1.12 µGy (1.26 µSv)	9.22 µGy	11.9 µGy (13.5 µSv)	15.0 µGy	7.01 µGy (7.93 µSv)	14.7 µGy

## 4 DISCUSSION

The spatial distribution of stray radiation around a CT gantry is often depicted as having a bilobed shape.^11^ However, as demonstrated in this work, in a simulated clinical scenario, the stray radiation distribution has a clover shape, as scattered radiation is attenuated not only by the gantry, but also by the patient and patient table.

The intensity of stray radiation was lowest at location T (Figure 2), related to both attenuation of scattered radiation by the gantry and the distance from isocenter at this location. At a height of 125 cm measured stray radiation levels were constant as a function of distance from the gantry (Figure 3). Stray radiation intensity was higher at a height of 175 cm, and there was some falloff in radiation dose at heights of 150 and 175 cm, this difference may be caused by the configuration of radiation shielding installed in the gantry. Two primary factors affected doses at location T: the fact that the closest an individual can approach isocenter in this direction is about 1.75 m, and the inherent shielding value of the structure of the gantry and gantry console. For similar reasons, measured K_a_ was higher at location O: the absence of the console allows individuals to approach as close as 1 m from isocenter and there is presumably less inherent shielding in this area of the gantry, which opens to allow for the gantry to be positioned over the patient. Measured *K_a_* at location A was intermediate, again for the same reasons: personnel can position themselves within 1.5 m of isocenter at this location, and the patient and patient table provide some shielding from stray radiation. At the same distance from isocenter, stray radiation was lower at location A than at location O.

Previous studies of stray radiation from mobile CBCT have reported widely varying results, and are summarized here, with accompanying numerical comparisons in Table 3. Sans Merce et al.^12^ used an Alderson phantom and tissue‐equivalent plastic scintillation detector (AT1123, APVL, Saint‐Cyr‐sur‐Loire, France) to measure ambient dose equivalent [H*(10)] for a single 3D pelvic acquisition and reported H*(10) values measured on a radial grid at isocenter height, which was not specified. Foster et al.^13^ made a single measurement of stray radiation during a cadaver study using a 60 cc ionization chamber (10 × 6‐60; Radcal, Monrovia, California, USA) and a “standard dose setting” of 120 kV, 63 mA (total mAs not reported). Ford et al.^14^ measured stray radiation in the shadow of the gantry 180 cm from isocenter at a height of 91.5 cm using a high‐definition large patient protocol (120 kV, 64 mA, 480 mAs), a 451P‐RYR survey meter, and a Lucite block to simulate a patient. Cewe et al.^15^ used a PBU‐60 phantom, which was very similar to the phantom used in the present work, but without any BMI slabs, and an 1800 cc ionization chamber (10 × 6‐1800; Radcal, Monrovia, California, USA) to map the stray radiation distribution on a radial grid for a standard dose, large patient spine protocol (120 kV, 200 mAs). Pitteloud et al.^16^ performed stray radiation measurements on a radial grid when scanning a 55 kg, 160 cm long female CIRS phantom using a small patient pelvis protocol (120 kV, 125 mAs) and reported H*(10) measured using a tissue‐equivalent plastic scintillation detector (AT1123, APVL, Saint‐Cyr‐sur‐Loire, France) on an interpolated grid. Zhang et al.^17^ reported stray radiation doses measured using an extra‐large upper torso protocol (120 kVp, 320 mAs), the 32 cm CTDI phantom, and an 1800 cc ionization chamber (10 × 6‐1800; Radcal, Monrovia, California, USA). Other authors have reported measures of stray radiation during patient studies^18^, ^19^, ^20^ for which it is not possible to make direct comparisons to the present work.

**TABLE 3:** Numerical comparisons to previous studies.

Author	Closest point of comparison (*x*,*y*,*z*) (cm)	Location in this study (*x*,*y*,*z*) (cm)	Stray radiation (prior)	Stray radiation (current)	Within measurement uncertainty? (Yes/No)
Sans Merce et al. ^12^	(−56, −56, ns*)	(−55, −55, 125)	0.453 µSv/mAs	0.355 µSv/mAs	Yes
Foster et al. ^13^	(71, 71, ns*)	(70, 70, 125)	67.1 µGy^†^	65.3 µGy^†^	Yes
Ford et al. ^14^	(−180, 0, 91.5)	(−200, 0, 100)	0.0031 µGy/mAs	0.00164 µGy/mAs	No
Cewe et al. ^15^	(141, 141, 160)	(140, 140, 125)	0.0805 µGy/mAs	0.0464 µGy/mAs	No
Pitteloud et al. ^16^	(0, −150, ns*) (−200, 0, ns*) (0, 200, ns*) (180, 0, ns*)	(0, −150, 125) (−200, 0, 125) (0, 200, 125) (180, 0, 125)	0.16 µSv/mAs 0.024 µSv/mAs 0.038 µSv/mAs 0.080 µSv/mAs	0.0316 µSv/mAs 0.00232 µSv/mAs 0.0137 µSv/mAs 0.0247 µSv/mAs	No No No No
Zhang et al. ^17^	(200, 0, 160) (0, 200, 160) (−200, 0, 160) (0, −200, 160)	(200, 0, 175) (0, 200, 175) (−200, 0, 175) (0, −200, 175)	0.057 µGy/mAs 0.14 µGy/mAs 0.014 µGy/mAs 0.14 µGy/mAs	0.0227 µGy/mAs 0.0334 µGy/mAs 0.0145 µGy/mAs 0.0293 µGy/mAs	No No Yes No

*Measurement height listed as isocenter but distance not specified.

^†^mAs not specified, per scan dose reported; direction from isocenter not specified, average of all four directions used from current study.

The wide range of stray radiation levels reported in previous studies is intriguing. The causes of the observed differences are likely multifactorial, and may include phantoms used, geometries studied, experimental setup, dosimetric quantities measured, differing energy responses of the different dosimeters used, mobile CBCT equipment and software changes over time, and differences in baseline radiation output. Comparisons to Zhang et al.^9^ (Table 2) were particularly interesting, as *K_a_* measured in the shadow of the operator console was very similar but at the head and foot end of the table differed by about a factor of 4, which would be the expected pattern when scanning an object with a limited extent, such as a CTDI phantom, compared to a full anthropomorphic phantom.

The findings of this study have practical implications for radiation safety in the OR environment, with due consideration given to the local regulatory environment. The first is that personnel who are already wearing personal radioprotective equipment during a surgical procedure, for example, in procedures using fluoroscopic guidance, will be adequately protected during mobile CBCT imaging. Depending on workload, setup, size of the operating room, mix of patient sizes, and other factors, scrubbed‐in personnel located at distances of 2 m or greater from isocenter (Figures 2 and 3) may not need to wear personal radioprotective equipment during mobile CBCT imaging, depending on the local regulatory environment. Assuming an equal mix of L and XL patients, the average stray radiation dose at 2 m from isocenter at location A was 12.8 µGy (14.5 µSv) per scan at a height of 150 cm (which represents the most conservative scenario for whole body dose) and K_a_ at a height of 175 cm (eye level) was 14.6 µGy per scan. At location O the stray radiation dose at 2 m from isocenter was 16.2 µGy (18.3 µSv) per scan at a height of 150 cm and K_a_ at a height of 175 cm was 15.0 µGy per scan. For comparison, the stray radiation dose at a height of 150 cm at location T was 4.86 µGy (5.49 µSv) per scan, *K_a_* at a height of 175 cm was 9.22 µGy per scan, and at a height of 125 cm was 4× lower. These doses are not so low that they can be ignored, but they are low enough to offer flexibility in radiation protection strategy. Further, radiation protection considerations may differ for each case, for example, sometimes personnel will already be wearing personal radioprotective equipment when fluoroscopy is being used. Radiation protection strategy might incorporate distance, including leaving the operating room during volumetric imaging; mobile shields to protect stationary staff such as anesthesia and neuromonitoring; standing behind personnel who are wearing personal radioprotective equipment; and it is very easy for personnel who are not scrubbed in to don and doff personal radioprotective equipment during volumetric imaging. This flexibility can afford the opportunity for some personnel (e.g., physicians and scrub techs) to remain in the room without breaking scrub, positioned at least 2 m away from isocenter on the console, head, or foot side of the patient, and not wear heavy personal radioprotective equipment for the duration of an hours‐long procedure when X‐rays are used for only a brief time. The effectiveness of these strategies can be assessed using personnel dosimetry records and corrective action taken if required, and OR staff training should address strategies for radiation protection.

## 5 CONCLUSIONS

When using a realistic simulated clinical imaging geometry, the stray radiation distribution around the O‐arm® O2 mobile CBCT imaging system had a clover shape. This study evaluated two realistic patient phantom sizes, used a real spine operating table, used both a regular and large acquisition field of view (FOV), and profiled the stray radiation distribution at multiple heights. Stray radiation was lowest at location T in the shadow of the gantry console, with a typical value for whole body dose of 1.77 µSv per scan measured at a height of 125 cm and a typical value for K_a_ at a height of 175 cm (eye lens level) of 9.22 µGy per scan. The maximum K_a_ measured 2 m or greater from isocenter was 18.3 µSv per scan at a height of 175 cm at location O, opposite the gantry console. These data can be used to inform location of personnel in the operating room, and stray radiation doses are low enough to allow use of several different radiation protection strategies, including personal radioprotective garments, mobile shields, or distance, to maintain occupational doses at acceptable levels.

## AUTHOR CONTRIBUTION

A. Kyle Jones designed the experiments, performed data acquisition, analyzed and interpreted the data, drafted and revised this manuscript, approved the published version, and is accountable for all aspects of the work.

## CONFLICT OF INTEREST STATEMENT

A. Kyle Jones received consulting fees and reimbursement for travel expenses from Medtronic, Inc. related to the present work.
